# Supplementary material for: Severity- and Time-Dependent Activation of Microglia in Spinal Cord Injury
Source: Int J Mol Sci. 2023 May 5;24(9):8294. doi: 10.3390/ijms24098294 (PMC10179339; doi:10.3390/ijms24098294)
Supplement: Supplementary file 1 [file ijms-24-08294-s001.zip › ijms-2339192-supplementary.pdf]

Supplementary Table S1. Primers and probes for RT-PCR

**Primer**	**Nucleotide sequence**
18S-TM-Forward	GCCGCTAGAGGTGAAATTCTTG
18S-TM-Reverse	CATTCTTGGCAAATGCTTTCG
Iba1-TM-Forward	ACCAGCGTCTGAGGAGCTAT
Iba1-TM-Reverse	AGGAAGTGCTTGTTGATCCC
CD209-TM-Forward	CCTCTCCCAAGTCAGCAGAA
CD209-TM-Reverse	CACACCATTTCACACAGCCA
TGF-β-TM-Forward	TGCTTCAGCTCCACAGAGAA
TGF-β-TM-Reverse	TCCAGGCTCCAAATGTAGGG
IL-6-TM-Forward	TGCCTTCTTGGGACTGATGT
IL-6-TM-Reverse	CTGGTCTGTTGTGGGTGGTA
CD40-TM-Forward	AGTGACAAACAGTACCTCCAAGG
CD40-TM-Reverse	TTGATTGAGTTCGCAGTGTCGG
TNF-α-TM-Forward	CCGTCCCTCTCATACACTGG
TNF-α-TM-Reverse	GTGCTCATGGTGTCCTTTCC
CCL-22-TM-Forward	TTCTTGCTGTGGCACTTCAG
CCL-22-TM-Reverse	CTCCTTCACGAAACGTGGTG
IL-1β-TM-Forward	GGGATGATGACGACCTGCTA
IL-1β-TM-Reverse	TGTCGTTGCTTGTCTCTCCT

Supplementary Table S2. Cytokine concentrations (pg/ml) of microglia supernatants on the model of SCI *in vitro*.

Groups		IL-1α	IL-2	GRO/KC	RANTES	G-CSF	MIP-1α	IL-6	MIP-3α
Medium		4,7±2,21	15,55±9,5	209,12±16	14,63±1,38	4,29±0,02	160,39±51,31	130,14±13,72	323,26±152,8
ISC		2,06±0,27	50,29±25,92	124,44±71,69	N/A	5,28±2,87	43,1±21,49#	24,23±9,38#	326,65±92,43
SCI 1.5	3 dpi	2,26±0,38	56,7±29,21	174,63±56,26	23,17±1,14	4,64±0,69	610,43±38,31*#**	4575,13±1197,74*#	434±162,21
	7 dpi	105,36±57,58*#	48,76±19,58	157,83±42,75	208,9±122,05*#	9,9±3,73#	71,76±17,99	17,87±6,69	496,61±183,17
	14 dpi	24,91±4,18	41,95±15,96	146,47±28,34	99,72±36,8*#	29,11±9,63*#	16,79±10,1	18,54±6,66	200,87±27,21
	60 dpi	102,15±16,81*#	798,56±38,75*#	1023,61±113,1*#	212,96±88,34*#	23,14±9,76*#	1184,54±154,39*#	8336,89±1230,99*#	1832,8±238,57*#
SCI 2.5	3 dpi	21,78±19,73	199,32±38,44*#	456,63±151,32*#	264,79±40*#	24,78±8,34*#	219,72±54,44*	4774,13±1126,68*#	880,55±101,99*#
	7 dpi	2,24±0,51	52,01±14,73	164,14±41,13	22,42±7,82	N/A	24,28±9,76	22,23±7,93	185,63±15,08
	14 dpi	36,91±1,22	33,44±12,18	488,44±73,37*#	N/A	N/A	42,85±20,25	2421,3±889,92*#	99,75±35,76*#
	60 dpi	2,07±0,69**	N/A	144,11±13,09	12,23±1,86	N/A	48,58±16,57**	514,47±85,49*#	90,99±30,09*#
SCI 4	3 dpi	2,19±0,66	522,79±109,18*#**	224,36±65	N/A	4,3±2,47	500,23±58,66*#	2127,49±582,49*#**	136,99±14*#
	7 dpi	112,09±28,69*#	890,93±461,75*#**	652,1±43,54*#**	449,94±169,32*#	57,22±9,63*#	673,1±88,26*#**	8084,12±83,06*#**	534,74±105,58
	14 dpi	78,54±19,81	628,53±84,64*#**	871,41±99,93*#**	392,09±114,93*#	25,88±9,57*#	541,44±77,79*#**	6070,33±1110,52*#**	403,71±61,93
	60 dpi	131,08±22,43*#	N/A	1010,24±88,33*#	325,57±45,66*#	14,11±3,24*#	1283,97±285,66*#	11596,18±3115,47*#	4270,88±1550,15*#**
**Groups**		**TNF-α**	**MCP-1**	**VEGF**	**IL-4**	**IL-13**	**IL-17**	**IL-10**	**IL-1β**
Medium		30,38±19,75	1402,96±189,58	19,10±5,13	N/A	N/A	4,4±3,1	N/A	8,84±5,22
ISC		32,4±18	664,66±18,1#	62,64±9,98#	14,93±10,62	N/A	2,36±0,64	N/A	N/A
SCI 1.5	3 dpi	39,49±18,85	908,39±350,53	53,9±43,82	15,05±2,56	N/A	6,51±0,72	N/A	2,26±0,38
	7 dpi	146,29±18,1*#	1262,66±539,74	49,1±23,4#	27,77±13,66	N/A	6,21±3,34	N/A	105,36±57,58*#
	14 dpi	73,86±32,81	2147,63±914,35*	53,45±19,78	40,03±21,28	23,61±2,43	4,65±2,42	384,36±2,6	24,91±4,18
	60 dpi	176,1±19,32*#	4574,61±1640,27*	1327,72±471,1*#	35,13±10,45	114,75±38,1	8,65±3,77	964,07±102,89**	102,15±16,81*#
SCI 2.5	3 dpi	35,75±13,13	3113,54±1831*	49,12±10,39#	21,98±8,08	N/A	5,92±2,23	522,94±150,46**	21,78±19,73
	7 dpi	30,06±9,77	658,15±322,58	51,31±16,13#	4,32±1,98	N/A	N/A	22,07±8,16	2,24±0,51
	14 dpi	N/A	2614±543*#	N/A	22,97±12,66	N/A	2,6±0,39	335,96±53,39	36,91±1,22
	60 dpi	N/A	279,42±137,35*#	26,67±8,84	N/A	N/A	N/A	137,57±14,34	2,07±0,69**
SCI 4	3 dpi	36,87±11,9	513,69±117,25#	32,51±19,45	N/A	N/A	N/A	N/A	2,19±0,66
	7 dpi	96,93±43,79	3330,6±1328,14*#	396,41±90,28*#**	26,81±11,26	N/A	8,98±2,51	1460,83±63,76**	112,09±28,69*#
	14 dpi	37,34±31,27	3456,79±467,94*#	1162,57±351,23*#	35,89±10,24	N/A	9,22±3,61	1410,03±171,2**	78,54±19,81
	60 dpi	191,66±66,49*#	5505,5±1254*#	2398,31±183,23*#**	53,97±13,63*	N/A	15,34±1,32*#	1747,53±334,55**	131,08±22,43*#

Groups		IL-5	IL-7	IL-12	IL-18	M-CSF	GM-CSF	IFN-γ
Medium		N/A	N/A	N/A	N/A	N/A	N/A	11,58±7,13
ISC		N/A	N/A	9,63±2,82	N/A	N/A	N/A	11,8±5,92
SCI 1.5	3 dpi	N/A	N/A	N/A	N/A	6,97±3,46	N/A	12,56±6,1
	7 dpi	11,98±7,2	19,7±10,44	N/A	N/A	N/A	N/A	12,14±6,61
	14 dpi	46,41±19,88	9,31±0,62	N/A	62,26±31,81	N/A	10,32±7,2	21,38±6,93
	60 dpi	54,76±25,55	56,66±16,43	N/A	N/A	N/A	25,44±8,02	22,52±4,29
SCI 2.5	3 dpi	25,26±13,83	N/A	N/A	N/A	N/A	8,43±2,65	14,97±4,05
	7 dpi	9,9±4,2	N/A	N/A	N/A	N/A	N/A	11,86±6,85
	14 dpi	32,85±16,7	N/A	N/A	59,33±27,77	N/A	N/A	13,44±7,44
	60 dpi	N/A	N/A	N/A	N/A	N/A	N/A	N/A
SCI 4	3 dpi	N/A	N/A	N/A	N/A	N/A	N/A	11,96±6,76
	7 dpi	N/A	N/A	N/A	N/A	N/A	N/A	2,48±1,71
	14 dpi	40,18±19,76	10,04±1,88	5,13±3,65	63,44±20,23	6,85±3,95	9,14±3,84	N/A
	60 dpi	58,92±24,68	32,41±6,49	N/A	152,04±49,54	5,5±1,42	10,25±1,87	25,13±9,16$^{*\#}$

N/A (not available) - protein concentrations are not detectable with the kit.

*P<0.05 as compared with ISC group. $^{\#}$P<0.05 as compared with Medium group. **P<0.05 as compared with other values at the appropriate time after SCI.

Significant difference is indicated for detectable values.
